# Editorial: Roles of Tumor-Recruited Myeloid Cells in Immune Evasion in Cancer

**DOI:** 10.3389/fimmu.2021.749605

**Published:** 2021-08-27

**Authors:** Sergei Kusmartsev, Paolo Serafini, Srinivas Nagaraj Bharadwaj, Marcin Kortylewski

**Affiliations:** ^1^Department of Urology, University of Florida, Gainesville, FL, United States; ^2^Department of Microbiology and Immunology, University of Miami, Miami, FL, United States; ^3^Department of Internal Medicine, University of South Florida, Tampa, FL, United States; ^4^Department of Immuno-Oncology, Beckman Research Institute at City of Hope Comprehensive Cancer Center, Duarte, CA, United States

**Keywords:** tumor microenvironment, immune evasion, immune tolerance, MDSC (myeloid-derived suppressor cell), tumor-associated macrophage

During tumor formation and progression, tumors develop an immunosuppressive and tolerogenic microenvironment. Tumor-associated myeloid cells, including myeloid-derived suppressor cells (MDSCs) and tumor-associated macrophages (TAMs) represent one of the major immunosuppressive components in the tumor. Tumor-recruited myeloid cells contribute to tumor growth by exerting profound immunosuppressive effects through inhibition of adaptive and innate anti-tumor immune responses, stimulation of tumor angiogenesis, and remodeling extracellular matrix. This Research Topic aims to provide a comprehensive overview of the tumor-recruited myeloid cell subsets, describe the immune function of MDSCs, TAMs, tumor-associated neutrophils, and review the novel approaches targeting myeloid cells to enhance anti-tumor immunity and improve the efficacy of cancer immunotherapy. Our collection of 12 manuscripts consists of 2 Original Research papers, 3 Mini-Reviews, and 7 Reviews are summarized below. This Research Topic covers different aspects of MDSCs biology, including their generation and mobilization in cancer, mechanisms of tumor-associated immune suppression, and their contribution to resistance to immunotherapy. Also, it highlights the novel approaches for targeting MDSCs in cancer.

## Generation and Mobilization of MDSC in Cancer

The increased accumulation of MDSCs in both peripheral blood and tumor tissues is very well documented for multiple cancer types in both experimental and clinical settings. Myeloid cell recruitment into tumors is associated with enhanced tumor-induced myelopoiesis and inflammation, which drives mobilization of myeloid cells to the tumor site. Tumors are inherently pro-inflammatory, with infiltrating myeloid cells thought to be critical for tumor development, maintenance, and progression. As demonstrated in experimental and clinical studies, both chronic and acute inflammation can drive tumorigenesis of different origins (1–4). Despite our still limited knowledge of cancer-specific mechanisms of generation and tumor recruitment, it is clear that bone marrow and spleen are major sources of MDSCs. Wu et al. concisely reviewed experimental and clinical studies highlighting the mechanisms of tumor-mediated stimulation of myelopoiesis in tumor hosts with a major focus on the spleen, the major site of extramedullary hematopoiesis in the cancer setting. The authors summarize the distinct mechanisms, functional specialization, and clinical relevance of cancer-associated myeloid cell generation in the spleen and its potential as a novel therapeutic target. They also provide insight into mechanisms of tumor-dependent development of MDSCs from hematopoietic stem cells and myeloid cell progenitors and highlight the roles of specific hematopoietic cytokines and tumor-derived factors in this process. Karin reviewed the roles of specific chemokine receptors and their ligands in cancer-associated multistep MDSC mobilization. Although myelopoiesis is coordinated by multiple cytokines and transcription factors, mobilization is selectively directed by chemokine receptors and may differ between M-MDSC and PMN-MDSC. These myeloid cells may then undergo further expansion at these secondary lymphatic organs and then home to the tumor site. Thus, mobilization of MDSCs from bone marrow to the blood is directed by specific chemokine receptors such as CCR2 for monocytic MDSCs *via* CCR2-specific ligand CCL2, and CCR5 for the PMN-MDSCs *via* CLL3, CCL4, or CCL5 ligands. It should be noted that other chemokine-mediated signaling pathways including CXCL/CXCR2 also contribute to the tumor recruitment of myeloid cells and that signal integration between different chemokines may also play an important role in their differentiation and function.

## Myeloid Cells in the Tumor Microenvironment

While PMN-MDSCs are relatively short-living cells, monocytic MDSCs upon entering tumor tissue frequently differentiate into immunosuppressive tumor-associated macrophages (TAMs) and can function there for a long time. Wu and Zhang summarize the broad role of TAMs and TANs (tumor-associated neutrophils) in cancer, provide detailed information on how these cells contribute to the growth of primary and metastatic tumors, and discuss their clinical relevance. Davidov et al. review the recent advances in data science, including bioinformatics, single-cell RNA sequencing (scRNAseq) and mass cytometry, which have enabled the development of novel approaches to explore the myeloid cells in the tumor microenvironment. These methods may provide a greater understanding of the mechanisms of tumor-associated immune suppression and tolerance, differentiation and polarization of myeloid cells in the tumor microenvironment, and interaction of tumor-infiltrating myeloid cells with immune cells, tumor cells, and stromal compartment. Zwing et al. provide a detailed analysis of spatial organizational patterns of tumor-associated myeloid cells and T cells in tumor tissues obtained from 74 previously untreated patients with colorectal cancer. This study combined the digital image-based analysis, including cell density, cell-to-cell distance, and spatial overlap, with gene expression profiling to link the tumor spatial features with the biological function of tumor-infiltrating immune cells. In these patients, MDSCs seem to accumulate mostly at the invasive tumor edge and are strictly associated with CD8+T cells. This confirmed previous findings from Weed et al. in a limited number of patients with HNSCC and suggests that myeloid cells may provide a physical barrier to exclude CTL from the tumors.

Currently, patient stratification models focus mostly on the tumor-infiltrating CD8^+^ T cells in tumor tissue. However, data provided by the authors, clearly suggest that both numbers of CD8^+^ T cells and the spatial relationship between myeloid and T cells should be taken into account for the immune-based patient classification and stratification. Additionally, similar analyses should be performed after therapy to evaluate how anti-cancer treatment modulate MDSC and T cell infiltration.

## Mechanisms of Immune Suppression and Immune Resistance Mediated by MDSC

Several molecular mechanisms deployed by cancer-associated myeloid cells mediate the inhibition of innate and adaptive anti-tumor immune response, thus promoting immune evasion. The review by Grzywa et al. focused specifically on the role of myeloid cell-derived arginase (ARG) in the regulation of cancer immunity. ARG expression is substantially elevated in myeloid cells in cancer and mitigates antitumor response *via* multiple mechanisms. Authors provide detailed overview of the biochemistry and metabolism of arginase and its substrate L-arginine in tumors and in tumor-associated myeloid cells. ARG-expressing myeloid cells strongly inhibit T cell proliferation by impairing CD3 zeta chain expression, and this effect could be reversed by supplementation of L-arginine or by small molecule arginase inhibitors. Lebegge et al. focused on multiple innate immune mechanisms by myeloid cells that contribute to cancer immunotherapy resistance and promote tumor growth. The mechanisms include the release of pro-inflammatory mediators, neutrophil degranulation and respiratory burst, neutrophil extracellular trap formation, tissue pathogen, and damage recognition mechanisms, and others. Daveri et al. summarize various roles of microRNA in shaping myeloid cell-mediated resistance to the cancer immunotherapy. Small non-coding RNA molecules, the microRNAs (miR) contribute to myeloid cell regulation at different levels, including cell metabolism and immune function, as well as affecting MDSC differentiation and skewing their phenotype. MiR expression in myeloid cells can be indirectly induced by tumor-derived factors or through direct miR import *via* extracellular vesicles. Indeed, extracellular vesicles are becoming important factors that regulate both MDSC differentiation and function.

## MDSC as Biomarkers for Response to the Cancer Immunotherapy

The increased presence of immunosuppressive cells in patient’s peripheral blood and tumor tissue may affect the response of cancer patients to immunotherapy. Peranzoni et al. reviewed the existing evidence of the relation between myeloid cell subsets and the response of cancer patients to the treatment with immune checkpoint inhibitors. The authors propose that circulating and tumor-infiltrating myeloid cell populations can be used as predictive biomarkers for immune checkpoint inhibitors in different human cancers, both at baseline and on treatment. Thus, in patients with advanced melanoma, treated with a combination of anti-PD1 antibody and a multi-peptide vaccine, the M-MDSC expansion in peripheral blood was associated with poorer response to the immunotherapy. The authors also note that given the plasticity of myeloid cells and the differences in the microenvironment among tumors, the phenotype of this lineage can greatly vary. Therefore, the simple abundance of CD68 cells, classically considered to represent macrophages, is thus rarely informative, while the functional orientation of myeloid cells by multi-parameter IHC, flow cytometry, or RNA sequencing allows defining a clearer relationship between the distinct subsets and the clinical outcome. This review also highlights myeloid cell plasticity and how treatments can induce the appearance of different immune evasion mechanisms.

## Targeting MDSC in Cancer

Recent years have been marked by significant progress in developing, clinical testing, and validation of new immunotherapeutic agents for cancer therapy, including immune checkpoint inhibitors, engineered immune cells, and novel cancer vaccines. However, the clinical efficacy of cancer immunotherapy is limited due to tumor-associated immune suppression and immune tolerance. Therefore, targeting MDSCs holds a great potential to boost the anti-tumor immune response and produce a more powerful therapeutic effect than immunotherapy alone. de Cicco et al. reviewed recent progress in the development of novel strategies for targeting MDSCs in cancer such as: (i) depletion of MDSC populations; (ii) prevention of MDSCs recruitment and/or migration to the tumor site; (iii) attenuation of immune suppression in cancer by targeting specific molecular pathways that are involved in MDSC-mediated inhibition of anti-tumor immune response; iv) promoting the differentiation of MDSCs into mature non-suppressive myeloid cells like M1-macrophages or dendritic cells. The heterogeneity of these myeloid cells makes their identification in human cancer very challenging. The authors suggest that since phenotype and mechanisms of action of MDSCs appear to be tumor-dependent, it is important to accurately characterize the precise MDSC subsets that have clinical relevance in each tumor environment to more efficiently target them. Also, the authors provide the specific characteristics of immunosuppressive myeloid cell subsets detected in several human cancers including melanoma, breast cancer, prostate cancer, colorectal cancer, hepatocellular carcinoma, and lung cancer. Alban et al. demonstrated that monocytic MDSCs in glioblastoma express high levels of CD74, which serves as a cognate receptor macrophage migration inhibitory factor (MIF). Targeting of MDSCs with ibudilast, a MIF-CD74 interaction inhibitor, resulted in a reduction of the production of monocyte chemoattractant protein 1 (MCP1, CCL2) and stimulated the expansion of CD8 T cells. However, using ibudilast as a single agent for the treatment of the experimental model of glioblastoma did not improve the survival rate. Sieminska and Baran reviewed targeting MDSCs in colorectal cancer. Since survival and expansion of MDSCs is regulated by PGE2, administration of COX2 or MPGES1 inhibitors in animals with experimental tumors results in a reduction of MDSCs and inhibition of tumor growth.

In conclusion, our Research Topic underscores the diverse roles of tumor-associated myeloid cell subsets in in immune evasion and the active suppression of anticancer immune responses. At the same time, this manuscript collection clearly indicates that there is still much to discover about the novel MDSC markers, cancer-specific mechanisms of MDSCs generation, and molecular pathways that control differentiation and polarization of recruited myeloid cells in tumor tissue.

## Author Contributions

All authors listed have made a substantial, direct, and intellectual contribution to the work, and approved it for publication.

## Funding

This work was supported by Fund 1923 to SK, SCCC Translational Science Grant to PS and by the National Cancer Institute/National Institutes of Health award number R01CA215183 and the Department of Defense grant W81XWH1910852 to MK.

## Conflict of Interest

SK is a founder of the K-Lab Therapeutics. PS is a founder and shareholder of ABTvax. MK is a founder of iSTAT Therapeutics and a scientific advisor to Scopus Biopharma. MK is a co-founder of Duet Therapeutics, shareholder and scientific advisor to Scopus Biopharma. PS is co-founder of ABTvax and scientific founder of Wink therapeutics.

The remaining author declares that the research was conducted in the absence of any commercial or financial relationships that could be construed as a potential conflict of interest.

## Publisher’s Note

All claims expressed in this article are solely those of the authors and do not necessarily represent those of their affiliated organizations, or those of the publisher, the editors and the reviewers. Any product that may be evaluated in this article, or claim that may be made by its manufacturer, is not guaranteed or endorsed by the publisher.
